# Health care utilization and cost differences across cognitively‐defined Alzheimer’s disease subgroups

**DOI:** 10.1002/alz.089511

**Published:** 2025-01-09

**Authors:** Norma Coe, Chuxuan Sun, Lindsay White, Lily N Shapiro, Janelle S Taylor, Paul K. Crane

**Affiliations:** ^1^ University of Pennsylvania, Philadelphia, PA USA; ^2^ Kaiser Permanente Washington Health Research Institute, Seattle, WA USA; ^3^ University of Toronto, Toronto, ON Canada; ^4^ University of Washington, Seattle, WA USA

## Abstract

**Background:**

Existing studies on the health care utilization and costs associated with Alzheimer’s disease (AD) have treated individuals with AD as a homogeneous group, though recent evidence suggests individuals with AD may be classified into biologically distinct subgroups with differing genetic and clinical profiles. The objective of our study is to examine differences in healthcare utilization and costs across cognitively defined AD subgroups.

**Method:**

We utilize data from the Adult Changes in Thought (ACT) study (1994 – 2020), a population‐based longitudinal study of aging and the incidence of and risk factors for dementia. We focus our study on individuals who developed incident AD and classify these individuals into one of six cognitively‐defined AD subgroups using previously described methods. We identify a sex‐ and birth year‐matched set of controls using a many‐to‐one matching method. Controls are assigned an index date equal to the AD onset date for their matched AD case. We examine utilization and costs in the year preceding AD onset and in the three years following. Our utilization outcomes include number of days in a month spent in a hospital inpatient, intensive care unit, or skilled nursing facility setting, and number of emergency department visits. We also examine monthly total health care costs and component costs, including outpatient, hospital inpatient, skilled nursing facility, and pharmacy costs. We utilize repeated measures generalized estimating equations to estimate health care utilization associated with each AD subgroup. To estimate the incremental costs associated with each subgroup, we use the Basu and Manning cost estimator.

**Result:**

We find significant utilization and costs differences across the cognitively‐defined AD subgroups, driven primarily by differences in the use of hospital inpatient and skilled nursing facility services. We also find the highest utilization and costs among the group of individuals with substantial relative impairments across multiple cognitive domains.

**Conclusion:**

Studies on the health care utilization and costs associated with AD miss important heterogeneity by examining individuals with AD in the aggregate. Our study suggests that individuals in the cognitively‐defined AD subgroups have distinct health care utilization and cost patterns leading up to and following AD onset.

